# Annual acknowledgement of manuscript reviewers

**DOI:** 10.1186/1423-0127-20-15

**Published:** 2013-03-21

**Authors:** Wen-Chang Chang

**Affiliations:** 1 Taipei Medical University, Taipei, Taiwan

## Abstract

**Contributing reviewers:**

The editors of *Journal of Biomedical Science* would like to thank all our reviewers who have contributed to the journal in Volume 19 (2012).

## 

Jee-Yin Ahn

South Korea

Mohammed Bahey-El-Din

Egypt

Shashi Bala

United States of America

Krishnaswamy Balamurugan

India

Riyaz Basha

United States of America

Kayla Bayless

United States of America

Valentina Bollati

Italy

Anna Breborowicz

Poland

Albert Breier

Slovakia

Chandana Buddhala

United States of America

Peter Celec

Slovakia

Chia-Hsin Chan

United States of America

James Yi-Hsin Chan

Taiwan

Nei-Li Chan

Taiwan

Samuel H.H. Chan

Taiwan

Alice Y.W. Chang

Taiwan

Cheng-Fu Chang

Taiwan

Kuo-Wei Chang

Taiwan

Fu-Hsiung Chang

Taiwan

Jer-Wei Chang

Taiwan

Jang-Yang Chang

Taiwan

Ming-Fu Chang

Taiwan

Ming-Shi Chang

Taiwan

Nen-Chung Chang

Taiwan

Tsuchung Chang

Taiwan

Tien-Chun Chang

Taiwan

Chungming Chang

Taiwan

Zee-Fen Chang

Taiwan

Lee-Young Chau

Taiwan

Ben-Kuen Chen

Taiwan

Chia-Hsiang Chen

Taiwan

Chih-Cheng Chen

Taiwan

Chin-Tin Chen

Taiwan

Chao-Long Chen

Taiwan

Chung-Ming Chen

Taiwan

Jun-An Chen

Taiwan

Jin-Jer Chen

Taiwan

Jianbo Chen

United States of America

Jin-Chung Chen

Taiwan

Kuen-Feng Chen

Taiwan

Ku-Chung Chen

Taiwan

Shu-Ching Chen

Taiwan

MinHuey Chen

Taiwan

Rita Chen

Taiwan

Ruey-Hwa Chen

Taiwan

Yaomin Chen

United States of America

Yi-Jen Chen

Taiwan

Yi-Rong Chen

Taiwan

Ann-Li Cheng

Taiwan

Jason Chia-Hsien Cheng

Taiwan

Tzu-Hurng Cheng

Taiwan

Yijuang Chern

Taiwan

Chin-Wen Chi

Taiwan

Ya-Hui Chi

Taiwan

Bor-Luen Chiang

Taiwan

Cheng-Ting Chien

Taiwan

Kuo-Liong Chien

Taiwan

Alice Chien Chang

Taiwan

Lih-Chu Chiou

Taiwan

Ing-Ming Chiu

Taiwan

Jeng-Jiann Chiu

Taiwan

Yung Hyun Choi

South Korea

Il Whan Choi

South Korea

Chen-Kung Chou

Taiwan

Tz-Chong Chou

Taiwan

Chi-Jen Chu

Taiwan

Lee-Ming Chuang

Taiwan

Shuang-En Chuang

Taiwan

Wan-Long Chuang

Taiwan

Yao-Chung Chuang

Taiwan

Chih-Pin Chuu

Taiwan

Julie Crockett

United Kingdom

Zen-Kong Dai

Taiwan

Giuseppe Damante

Italy

Pranab Kumar Das

Netherlands

Heleen de Koning

Netherlands

Yoshiharu Deguchi

Japan

Henri-Jacques Delecluse

Germany

Sharmistha Dey

India

Gilbert Donders

Belgium

Anna Dongari-Bagtzoglou

United States of America

Dipak Dube

United States of America

Abdoelwaheb El Ghalbzouri

Netherlands

Irina Elovaara

Finland

Kang Fang

Taiwan

Charlotte Farin

United States of America

Simon Fricker

United States of America

Wen-Mei Fu

Taiwan

Zhen Fu

United States of America

Teruhiko Fujii

Japan

Masahiro Fujimuro

Japan

Yasuhisa Fukui

United Kingdom

Manish Gandhi

United States of America

Wei Ge

Hong Kong

Po-Wu Gean

Taiwan

Sarah Gilbert

United Kingdom

Wendy Goodman

United States of America

Jih-Hwa Guh

Taiwan

Cheng Guo

China

Fiorella Gurrieri

Italy

Bert Jan Haijema

Netherlands

Kyudong Han

United States of America

Michal Hetman

United States of America

Chuang Ye Hong

Taiwan

Pei-Wen Hsiao

Taiwan

Ching-Hua Hsieh

Taiwan

Po-Shiuan Hsieh

Taiwan

Sen-Yung Hsieh

Taiwan

Hsin-Ling Hsu

Taiwan

Tsai-Ching Hsu

Taiwan

Kuei-Sen Hsu

Taiwan

John Hsu

Taiwan

Cheng-Po Hu

Taiwan

Eagle Yi-Kung Huang

Taiwan

Kun-Lun Huang

Taiwan

Shiu-Feng Huang

Taiwan

Shih-Ming Huang

Taiwan

Tze-Sing Huang

Taiwan

Tur-Fu Huang

Taiwan

Xupei Huang

United States of America

Yi-You Huang

Taiwan

Chien-Fu Hung

United States of America

Hui-chih Hung

Taiwan

Wen-Chun Hung

Taiwan

Li-Man Hung

Taiwan

Jan-Jong HUNG

Taiwan

Lih-Hwa Hwang

Taiwan

Sam Hwang

United States of America

Satoru Iida

Japan

Kouichi Itoh

Japan

Steven Jacobson

United States of America

Rienk Jeeninga

Netherlands

Ching-Chuan Jiang

Taiwan

Yuh-Shan Jou

Taiwan

Shyr-Te Ju

United States of America

Chi-Chang Juan

Taiwan

Yiu Wing Kam

Singapore

Shinji Kamada

Japan

Hong-Yo Kang

Taiwan

Keiko Kawamoto

Japan

Ushio Kikkawa

Japan

Jaehyup Kim

United States of America

Sung Kim

South Korea

Takuya Kishi

Japan

Shigetaka Kitajima

Japan

Yu Ru Kou

Taiwan

Joost Kreijtz

Netherlands

Jan Krijt

Czech Republic

Julia Kuliwaba

Australia

John Kung

Taiwan

Szu-Hao Kung

Taiwan

Ching-Chuan Kuo

Taiwan

Yung-Ting KUO

Taiwan

Sheng-Chu Kuo

Taiwan

Michael Ming-Chiao Lai

Taiwan

Scott Langevin

United States of America

Roberto Latini

Italy

Hyuk-Joon Lee

South Korea

Hsuan-Shu Lee

Taiwan

Tzong-Shyuan Lee

Taiwan

Yann-Jinn Lee

Taiwan

Yi-Hsuan Lee

Taiwan

Guey-Jen Lee-Chen

Taiwan

Jan Lehotsky

Slovakia

Michael Lenardo

United States of America

Giovanni Levi

France

Ruey-Ming Liao

Taiwan

Yung-Feng Liao

Taiwan

Ching-Ling (Ellen) Lien

United States of America

Teng-nan Lin

Taiwan

Ching-Yuang Lin

Taiwan

Kwang-Huei Lin

Taiwan

Shih-Hua Lin

Taiwan

Chun Liang Lin

Taiwan

Jung-Yaw Lin

Taiwan

Shuei-Liong Lin

Taiwan

Ya-wen Lin

Taiwan

A. Maan-Yuh Lin

Taiwan

Wan-Wan Lin

Taiwan

Yu-Hsin Lin

Taiwan

Yu-yi Lin

Taiwan

Pin Ling

Taiwan

Thai-Yen Ling

Taiwan

Jun-Yang Liou

Taiwan

Chun-Jen Liu

Taiwan

Fu-Tong Liu

United States of America

Ko-Jiunn Liu

Taiwan

Ping-Yen Liu

Taiwan

Shufeng Liu

United States of America

Shing-Hwa Liu

Taiwan

Yan Liu

United States of America

Yu-Chih Lo

Taiwan

Shao-Chun Lu

Taiwan

Da-Wen Lu

Taiwan

Sekhar Majumdar

India

Isao Matsuura

Taiwan

Yaa-Jyuhn Meir

Taiwan

Shi-Chuen Miaw

Taiwan

Sarah Milton

United States of America

Ryo Miyazaki

Switzerland

Susanne Modrow

Germany

Cláudia Moura

Brazil

Ruiling Mu

United States of America

Dafne Müller

Germany

Tomiyasu Murata

Japan

Takahiko Naka

Japan

Akio Nakashima

Japan

Britto Nathan

United States of America

David Naylor

United States of America

Tadamasa Ooka

France

Helen O'Shea

Ireland

Li-Mei Pai

Taiwan

Chunliu Pan

United States of America

Tai-Long Pan

Taiwan

Hongbo Pang

United States of America

Jong-Hwei Pang

Taiwan

Luis A Pardo

Germany

Pier Giuseppe Pelicci

Italy

Claudia A. Penafuerte-Diaz

Canada

Robert Peng

Taiwan

Serguei Popov

United States of America

Howard Prentice

United States of America

Yeong-Shiau Pu

Taiwan

Paul Pumpens

Latvia

Stanley Rapoport

United States of America

Rosemary Redfield

Canada

Rei-Cheng Yang

Taiwan

Steve Roffler

Taiwan

Thomas Rogers

United States of America

Jihane Romanos

Lebanon

Heiner Schaal

Germany

Wolfgang Schmidt

Austria

David Schneider

United States of America

C.-K. James Shen

Taiwan

Wei Shen

United States of America

Wen Shen

United States of America

Joen-Rong Sheu

Taiwan

Ming-Jyh Sheu

Taiwan

Feng-Shiun Shie

Taiwan

Chi-Chang Shieh

Taiwan

Chiaho Shih

Taiwan

Hsiu-Ming Shih

Taiwan

Shin-Ru Shih

Taiwan

Noriaki Shimokawa

Japan

Kou-Gi Shyu

Taiwan

Richard Siegel

United States of America

Kwok-Fai So

Hong Kong

Kadaba Sriprakash

Australia

Robert Stackman

United States of America

Alexander Steinle

Germany

Chun-Kuei Su

Taiwan

Ming-Jai Su

Taiwan

Hong-Lin Su

Taiwan

Ih-Jen Su

Taiwan

Yeu Su

Taiwan

Dengyun Sun

United States of America

Yi Henry Sun

Taiwan

Ying Sun

United Kingdom

Man-Sun Sy

United States of America

Piotr Szefer

Poland

Ming-Hong Tai

Taiwan

You-Lin Tain

United States of America

Bertrand Tan

Taiwan

Tse-Hua Tan

Taiwan

Chih-Hsin Tang

Taiwan

Chih-Hsin Tang

Taiwan

Ming-Jer Tang

Taiwan

Rui Tao

United States of America

Hongyu Tian

United States of America

Shih-Feng Tsai

Taiwan

Chien-Sung Tsai

Taiwan

Meei-Ling Tsaur

Taiwan

Ching-Jiunn Tseng

Taiwan

Shun-Fen Tzeng

Taiwan

Takafumi Uchida

Japan

Rainer G. Ulrich

Germany

Robert Ulrich

United States of America

Thijs van Montfort

Netherlands

Cecilia Vazquez-Rovere

Argentina

Jia-Yi Wang

Taiwan

Tian-Li Wang

United States of America

Feng-Sheng Wang

Taiwan

Ju-Ming Wang

Taiwan

Naoki Watanabe

Japan

Douglas Watts

United States of America

Jenny Wei

United States of America

Vincent Wong

Hong Kong

Chin-Chen Wu

Taiwan

Gwo-Jang Wu

Taiwan

Hua-Lin Wu

Taiwan

Han-Chung Wu

Taiwan

Jaw-Ching Wu

Taiwan

Josh Wu

Taiwan

Kwan-Dun Wu

Taiwan

Kou-Juey Wu

Taiwan

Kenneth K. Wu

Taiwan

Sheng-Nan Wu

Taiwan

Kay LH Wu

Taiwan

Chia-Chao Wu

Taiwan

T.-C. Wu

United States of America

Ting-Feng Wu

Taiwan

Yang-Chang Wu

Taiwan

Kemin Xu

United States of America

Bo-Shiun Yan

Taiwan

Norimoto Yanagawa

United States of America

Cheryl Yang

Taiwan

Chuen-Mao Yang

Taiwan

Ning-Sun Yang

Taiwan

Wei-Shiung Yang

Taiwan

San-Nan Yang

Taiwan

Hung-I Yeh

Taiwan

Shiou-Hwei Yeh

Taiwan

B. Linju Yen

Taiwan

Hsueh-Chi Sherry Yen

Taiwan

Mao-Hsiung Yen

Taiwan

Ruoh-Fang Yen

Taiwan

Tzu-Chen Yen

Taiwan

Ming Yin

China

Hon-Kan Yip

Taiwan

Tai-Horng Young

Taiwan

Hsin-Su Yu

Taiwan

Ming-Lung Yu

Taiwan

Xiaocong Yu

United States of America

Pawel Zagrodzki

Poland

Yaxue Zeng

United States of America

Dekai Zhang

United States of America

Ming Zhang

United States of America

Mei-Yun Zhang

Hong Kong

Yongsheng Zhou

China

Yong Zhu

United States of America

